# Reversible Vision Loss With Pituitary Microadenoma Following PRP Injection for Hair Growth: A Rare Case Report and a Mini‐Review of the Literature

**DOI:** 10.1002/ccr3.71191

**Published:** 2025-10-07

**Authors:** Fazeela Bibi, Ahmad Sanan, Muhammad Hamza, Muhammad Talha Rehman Sherani, Sultan Salahuddin, Khalil El Abdi, Said Hamid Sadat

**Affiliations:** ^1^ Jinnah Medical and Dental College Karachi Pakistan; ^2^ Khyber Medical College Peshawar Pakistan; ^3^ Saidu Medical College Swat Pakistan; ^4^ Sandeman Provincial Hospital Quetta Pakistan; ^5^ Faculty of Medicine and Pharmacy of Rabat Mohammed V University Rabat Morocco; ^6^ Kabul University of Medical Science Abu Ali Ibn Sina Kabul Afghanistan

**Keywords:** hair regrowth, pituitary microadenoma, platelet‐rich plasma, skin revitalization, vision loss

## Abstract

Platelet‐rich plasma (PRP) therapy, while widely utilized for its favorable safety profile, carries a recognized risk of catastrophic, irreversible vision loss, traditionally attributed to iatrogenic vascular embolization following facial injections. We present the first documented case of *reversible*, subacute vision loss following PRP injections to the *scalp*, a finding that fundamentally challenges this established paradigm. A 22‐year‐old female presented with unilateral central scotomas one month post‐treatment, with investigations revealing macular edema and an incidental, non‐compressive pituitary microadenoma. In contrast to direct embolic occlusion, we postulate a novel pathophysiological mechanism: a systemic surge of PRP‐derived growth factors (e.g., VEGF, PDGF) triggered a transient, steroid‐responsive inflammatory or microvascular event in a pre‐sensitized neurovascular environment. The patient achieved complete visual recovery following high‐dose corticosteroid therapy, supporting an inflammatory etiology. This case serves as an unequivocal clinical directive that the risk of severe ocular complications from PRP is not confined to facial injections and can manifest systemically. It is imperative that the paradigm of PRP‐related risk be expanded and informed consent protocols revised to reflect the potential for inflammatory and microvascular sequelae, irrespective of the injection site.

**Summary**

- Caution is advised when considering PRP treatment due to its association with various side effects.
- A thorough evaluation of individual circumstances is necessary to carefully weigh the potential benefits against the risks, ensuring informed decision‐making and minimizing harm to optimize patient outcomes.

## 1 Introduction

Platelet‐rich plasma (PRP) is an autologous concentrate of platelets and their associated growth factors, produced by centrifuging a patient's own blood. This platelet‐rich fraction is then administered via injection or topical application for a range of therapeutic and aesthetic purposes [1]. It has emerged as a prominent treatment modality in regenerative medicine, with a growing body of evidence suggesting its beneficial role in hair regrowth [2, 3]. The therapeutic principle of PRP lies in its delivery of a rich milieu of fundamental protein growth factors—including platelet‐derived growth factor (PDGF), transforming growth factor‐β (TGF‐β), vascular endothelial growth factor (VEGF), and epithelial growth factor (EGF)—directly to the target tissue [4].

Over the past decade, PRP has been established as a valuable therapeutic tool in various specialties, including maxillofacial surgery, plastic surgery, orthopedics, and sports medicine. More recently, its application has expanded into dermatology and aesthetic medicine, where it is utilized for skin revitalization and facial rejuvenation [5, 6]. However, while its autologous nature mitigates the risk of allergic reactions or rejection [6], PRP therapy is not without potential side effects. Though often considered safe, rare but severe complications have been reported, the most devastating of which is vision impairment, including irreversible blindness [7].

## 2 Case History

A 22‐year‐old female with no significant prior medical history presented to an ophthalmologist with a chief complaint of decreased vision in her left eye, describing grayish spots in her central visual field. The visual loss was unilateral and painless. She further described the central scotoma as being composed of both black and gray spots, accompanied by a reduced perception of brightness.

On examination, her visual acuity was 6/24 in the left eye and 6/9 in the right eye. Her medical history was notable for a recent platelet‐rich plasma (PRP) treatment on the scalp for hair loss. The patient recalled an episode of transient, bilateral blurring of vision 10 days post‐PRP, which resolved spontaneously within 2 days. The onset of her current symptoms began 1 month after the scalp PRP procedure. The patient had no history of hypertension, diabetes mellitus, stroke, or multiple sclerosis. She denied any current medication use or a family history of autoimmune disease.

## 3 Differential Diagnosis, Investigations, and Treatment

**FIGURE 1:**
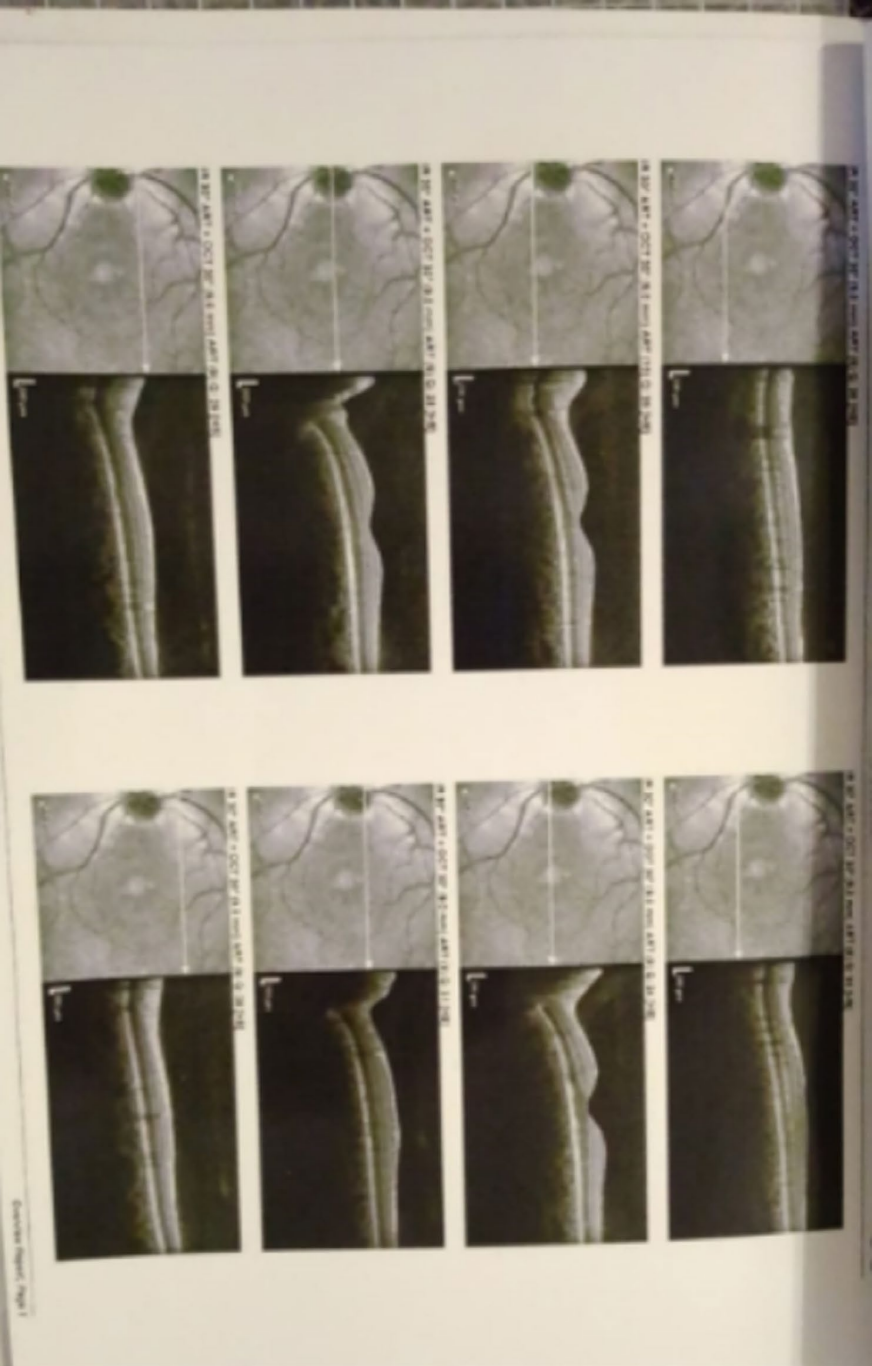
OCT optic disc.

**FIGURE 2:**
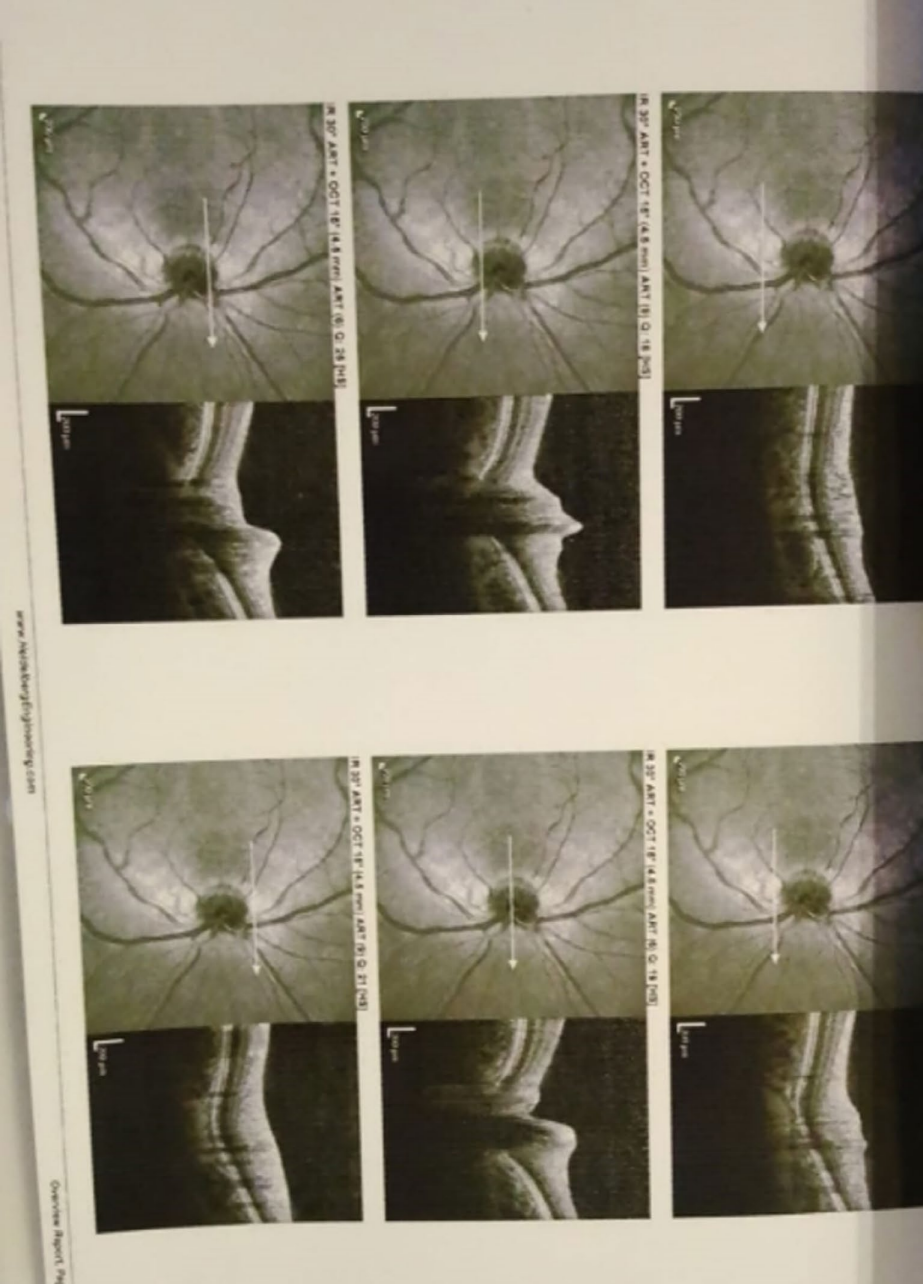
OCT optic disc.

**FIGURE 3:**
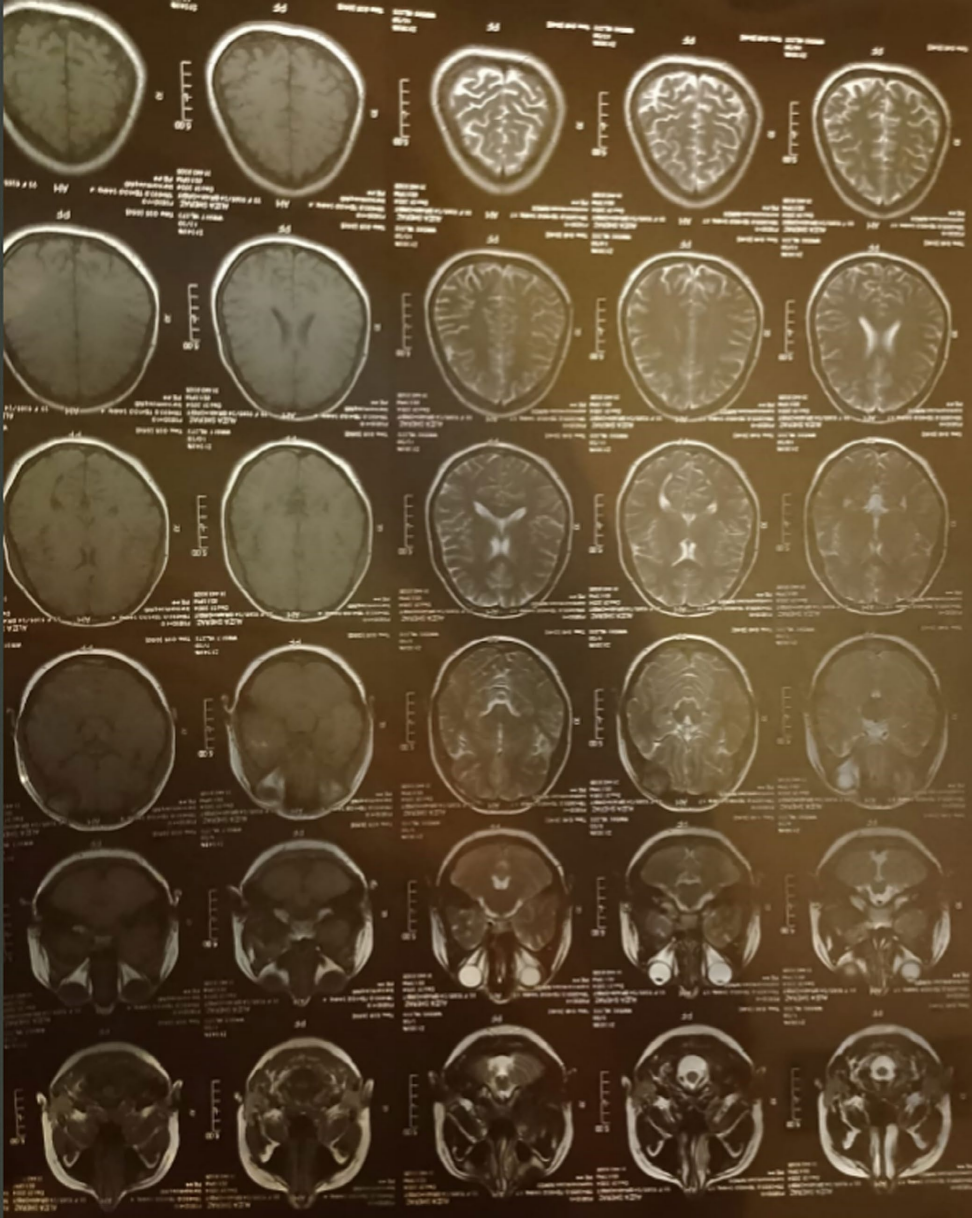
MRI axial view.

**FIGURE 4:**
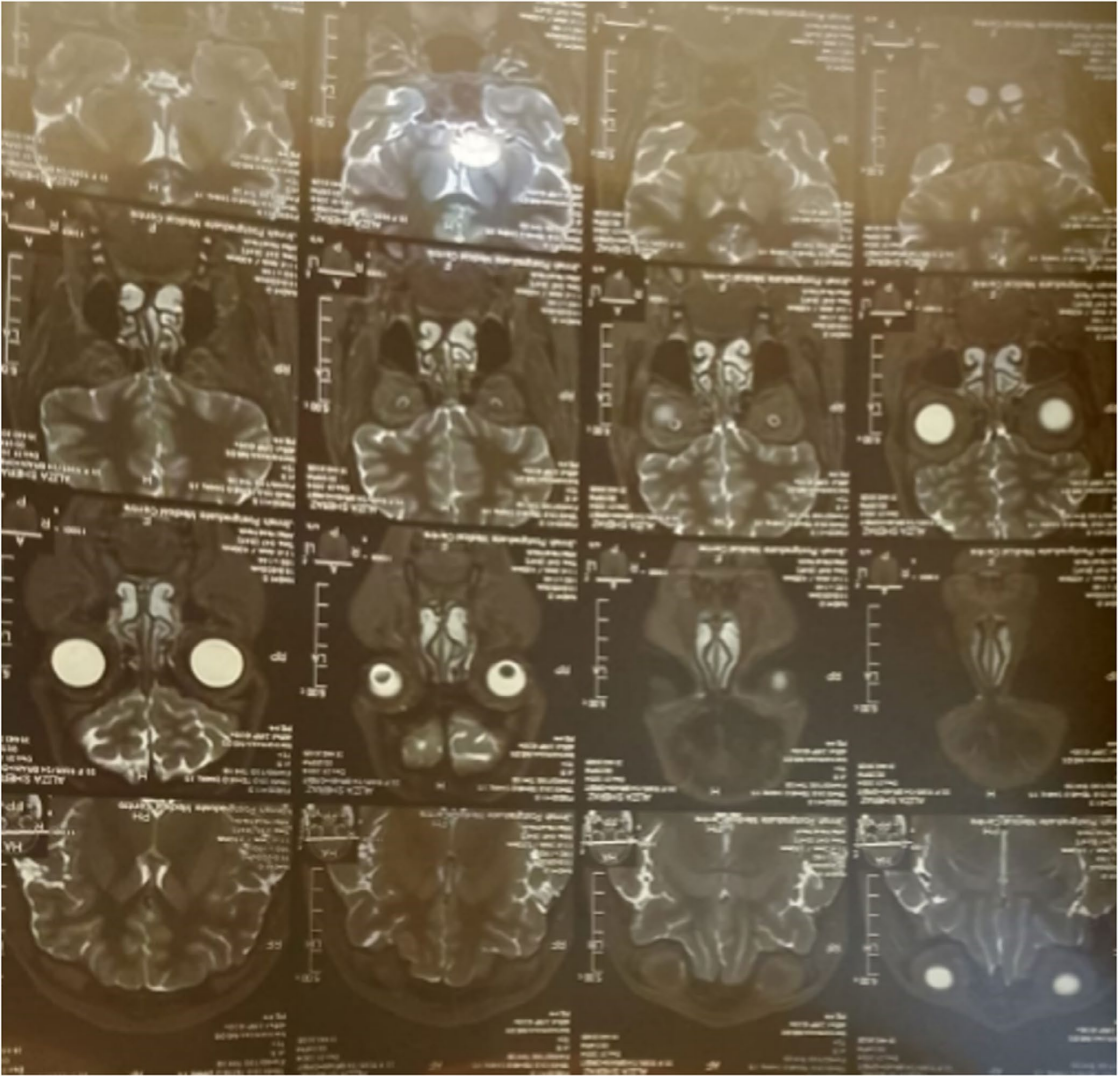
MRI coronal view.

**FIGURE 5:**
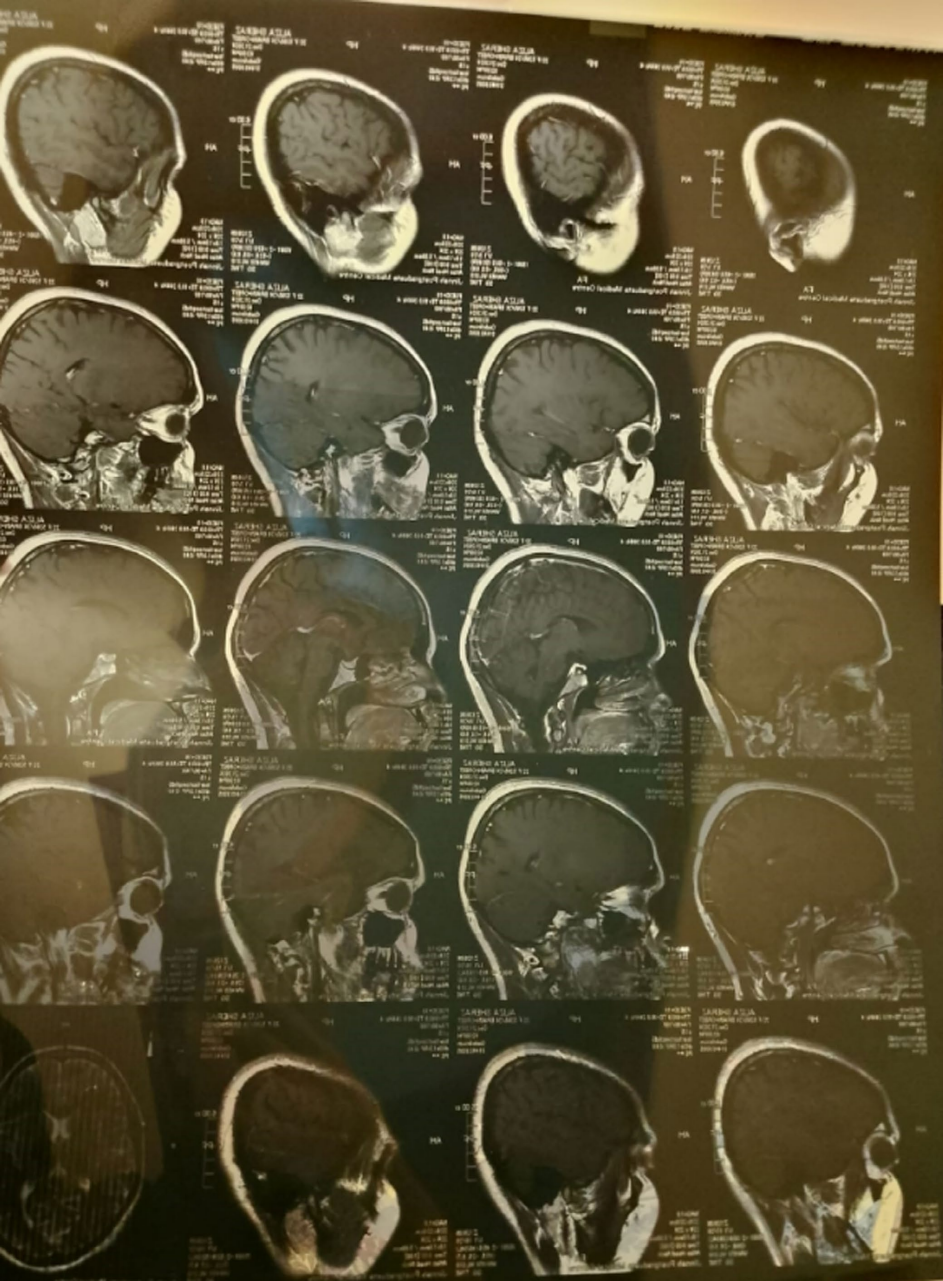
MRI side view.

An initial ophthalmologic evaluation included Optical Coherence Tomography (OCT) of the retina and retinal nerve fiber layer (RNFL), which revealed macular edema and borderline thinning in the temporal retinal sector (Figures 1 and 2). To investigate for a central etiology of the patient's visual symptoms, a brain MRI was performed. The MRI was unremarkable except for the incidental discovery of a pituitary microadenoma; critically, this lesion did not exert any mass effect on the optic chiasm (Figures 3, 4, 5). A comprehensive workup was subsequently initiated to assess the functionality of this pituitary incidentaloma. The patient reported no headaches, galactorrhea, or amenorrhea and exhibited no clinical features of acromegaly or Cushing's syndrome. Endocrinological assessment, including serum prolactin, IGF‐1, free T4, and cortisol levels, yielded results within normal limits, suggesting the adenoma was non‐functioning. Further serological testing was conducted to exclude systemic autoimmune or neuroinflammatory conditions as the cause of her optic neuropathy. An extensive panel, including tests for ANA, anti‐RNP, anti‐Ro, anti‐Scl‐70, anti‐Jo‐1, anti‐aquaporin‐4 (for NMOSD), and anti‐myelin oligodendrocyte glycoprotein antibodies, was entirely negative. Based on the clinical presentation and the exclusion of other identifiable pathologies, a presumptive diagnosis of inflammatory optic neuropathy was made. The patient was initiated on a course of high‐dose oral corticosteroids (Prednisolone), administered for 5 days, followed by a 3‐week maintenance dose (0.5 mg/kg/day) and a subsequent taper. She reported significant improvement in her vision within 10 days (timeline summarized in Table 1) of treatment initiation, with complete resolution of all symptoms documented at her 1‐month follow‐up.

**TABLE 1:** Clinical timeline of events.

Time point	Clinical event	Key findings/investigations	Intervention/treatment
Day 0	Scalp PRP injection for hair loss	N/A	Platelet‐rich plasma
Day +10	Onset of transient, bilateral blurred vision	Self‐reported by patient	None (resolved spontaneously within 2 days)
Day +30	Onset of unilateral central scotoma (left eye); patient presents to ophthalmologist	Visual acuity: 6/24 (L), 6/9 (R) OCT: Macular edema MRI: Incidental, non‐compressive pituitary microadenoma Labs: Negative autoimmune and endocrinology panels	Diagnostic workup
Post‐day +30	Presumptive diagnosis of inflammatory optic neuropathy made	Based on clinical picture and exclusion of other causes	High‐dose oral prednisolone initiated (5‐day course followed by taper)
Within 10 days of treatment	Patient reports significant visual improvement	N/A	Prednisolone course ongoing
One month post‐treatment Initiation	Complete resolution of all visual symptoms	Documented at follow‐up	Prednisolone course completed/tapered

## 4 Discussion

Platelet‐rich plasma (PRP) therapy has gained widespread adoption in aesthetic and regenerative medicine [2, 5], largely based on a favorable safety profile attributed to its autologous origin. However, this case report delineates a rare but severe complication—reversible vision loss following scalp injections—that challenges prevailing safety assumptions.

While PRP‐induced vision loss has been documented, existing reports (Table 2) are overwhelmingly linked to facial injections in highly vascularized regions [7, 8, 9, 11, 12, 13], where the mechanism is presumed to be iatrogenic vascular embolization leading to ophthalmic artery occlusion and irreversible ischemic injury [14, 15, 16]. Our case critically diverges from this paradigm: the injection site was the scalp, anatomically distant from orbital structures, and the clinical course featured a delayed, subacute onset with complete recovery, a presentation inconsistent with direct, large‐vessel occlusion.

**TABLE 2:** Review of similar case reports, PRP indications and their final visual acuity outcomes.

Patient demographics	PRP indication	Injection site	Time to vision loss	Eye/initial VA	Pain	Associated symptoms	Treatments (timeline)	Outcome/final VA
41 F, United Kingdom [7]	TMJ disorder	TMJ	Immediate	L: NLP	NR	NR	Pars plana vitrectomy; atropine gtt; dexamethasone gtt	No improvement; iris neovascularization
52 F, Venezuela [8]	Rejuvenation	R nasolabial fold; glabella	NR	R: NLP	Yes	Vomiting	NR	No VA follow‐up; at 1 month: cutaneous necrosis (forehead, peri‐orbital, cheek, right nasal ala)
48 F, Malaysia [9]	Rejuvenation	Glabella	Immediate	L: 20/60	Yes	Headache; glabellar hypoesthesia	3 h: ocular massage, hyperventilation, oral anti‐glaucoma; 9 h: timolol gtt, dexamethasone gtt, acetazolamide PO; D4: systemic steroids ×14 days	D4: L 20/200; M3: L 20/20 with residual RAPD, red desaturation, optic pallor, larger cup
50 F, Venezuela [8]	Rejuvenation	Forehead; glabella; R lateral canthus	Immediate	R: NLP	NR	Transient blue vision; headache; nausea; R ptosis; urinary urgency	NR	No follow‐up
6 F, Venezuela [8]	Rejuvenation (rhytides)	Forehead	NR	R: NLP	Yes	Dizziness; tinnitus; vomiting	NR	No follow‐up
49 F, USA [10]	Rejuvenation	Right glabella	Immediate	R: NLP	Yes	Nausea; ocular fullness	D1: massage; timolol gtt; brimonidine gtt; oral steroids; IV antibiotics	1 year: R NLP; glabellar scar; firm nodules
61 F, Venezuela [8]	Rejuvenation	Left glabella	Immediate	L: NLP	Yes	Dizziness; vomiting	NR	No VA follow‐up; at 8 months: L retinal detachment; glabellar scar
45 F, Italy [11]	Rejuvenation	Left glabella	Immediate	L: NLP	Yes	NR	D1: IV steroids; W1: enoxaparin injections	No improvement; preretinal hemorrhages (inferior temporal arcade); disc gliosis
37 F, USA [12]	Facial filler	Left glabella	Immediate	L: NLP	NR	Loss of consciousness; nausea; vomiting; dysarthria	NR	No improvement; choroidal infarction; enophthalmos; phthisis

The incidental discovery of a pituitary microadenoma introduces a novel and clinically significant variable. Although the absence of optic chiasm compression rules out a direct mass effect [17, 18], its presence prompts a compelling hypothesis: the systemic introduction of PRP‐derived growth factors may have triggered a pathological response in a pre‐sensitized neurovascular environment.

PRP is a potent cocktail of growth factors, including VEGF and PDGF [4, 19], which creates a powerful pro‐angiogenic and tissue‐remodeling milieu. Critically, these same growth factors, particularly VEGF, are the primary drivers of pathological neovascularization and vascular permeability in proliferative retinopathies and maculopathies. We postulate that a systemic surge of these factors could have interacted with the microvasculature of the optic nerve or pituitary region, unmasking a latent pathology.

Given this framework, the pathophysiology likely stems from an interconnected mechanism initiated by the systemic surge of pro‐angiogenic factors. It is plausible that high concentrations of PRP‐derived VEGF induced a state of transient microvascular dysregulation within the delicate circulation of the retina. This could lead to increased vascular permeability, resulting in the observed macular edema—a hallmark of maculopathy. This vascular leakage itself can trigger a secondary inflammatory cascade, a view strongly supported by the patient's clinical presentation with central scotomas, OCT findings of macular edema, and the known pro‐inflammatory potential of PRP injections [12, 20]. The definitive and rapid resolution of all visual symptoms with corticosteroid treatment provides robust evidence for this terminal inflammatory cascade, even if the initial trigger was vascular. This presentation should be distinguished from classic chronic proliferative retinopathy, which involves sustained neovascularization. In this case, the insult appears to have been a transient, acute event related to a temporary systemic bolus of growth factors, rather than a sustained pathological process, which explains both the subacute onset and the complete recovery. Ultimately, the subacute, steroid‐responsive clinical course is most consistent with an inflammatory optic neuropathy or a small‐vessel microvascular event rather than a catastrophic embolic occlusion.

In accordance with the WHO‐UMC criteria for causality assessment [21], the relationship between the scalp PRP procedure and subsequent optic neuropathy is classified as probable/likely, based on temporality, positive dechallenge, biological plausibility, and the exclusion of alternative causes. This case serves as a crucial clinical directive, demonstrating that the potential for vision‐threatening complications from PRP therapy is not confined to high‐risk facial injections. The paradigm of PRP‐related risk must be expanded to include systemic inflammatory and microvascular sequelae. Consequently, informed consent protocols should be updated to reflect this risk, regardless of the treatment area. Further research is imperative to elucidate the systemic impact of PRP‐derived cytokines and to establish definitive safety guidelines that account for these potentially serious neurological and ophthalmological adverse events.

## 5 Conclusion

This report documents the first known case of reversible vision loss following platelet‐rich plasma (PRP) therapy administered to the scalp, a finding that fundamentally expands the recognized spectrum of PRP‐related adverse events. The delayed, subacute clinical course and complete resolution with corticosteroid therapy critically differentiate this case from previously reported iatrogenic vascular occlusions, pointing instead toward an inflammatory or microvascular pathophysiology as the underlying mechanism. While the coexistence of a non‐compressive pituitary microadenoma was incidental, it raises provocative questions about the potential for systemically circulated PRP‐derived growth factors to unmask or exacerbate latent neurovascular pathologies. This case serves as an unequivocal clinical directive: the risk paradigm for PRP therapy must be broadened beyond direct embolic events to include systemic inflammatory and ischemic sequelae. Therefore, it is imperative that informed consent protocols are revised to explicitly include the risk of severe ocular complications, irrespective of the injection site. Ultimately, these findings highlight an urgent need for further research to elucidate the systemic biological impact of PRP and to establish evidence‐based safety guidelines that protect against these serious, unforeseen neurological adverse events.

## Author Contributions

**Fazeela Bibi:** formal analysis, resources. **Ahmad Sanan:** conceptualization, investigation, methodology. **Muhammad Hamza:** data curation, formal analysis, validation. **Muhammad Talha Rehman Sherani:** resources, writing – original draft. **Sultan Salahuddin:** writing – review and editing. **Khalil El Abdi:** drafting, editing, final review, and proofreading. **Said Hamid Sadat:** project administration.

## Consent

Written consent was taken from the patient. The patient is in contact with the authors.

## Conflicts of Interest

The authors declare no conflicts of interest.

## Data Availability

The data was taken from a patient who presented to our hospital; all data and references are publicly available on databases such as Pub‐med and Google Scholar.
